# The Burden of Cleft Surgery a 36-Year Reflection of Surgical Management of Children With Orofacial Clefts in South Australia

**DOI:** 10.1177/10556656251387961

**Published:** 2025-10-21

**Authors:** Emilija D Jensen, Gustavo Soares, Steven Cook, Markus Luke Seifert, Xiangqun Ju, Mark Moore, Rachel Roberts, Peter J Anderson, Lisa M Jamieson

**Affiliations:** 1 Australian Research Centre for Population Oral Health, Adelaide Dental School, 50071 the University of Adelaide, Adelaide, Australia; 2 Department of Paediatric Dentistry, Women's and Children's Hospital, North Adelaide, Australia; 3 Cleft and Craniofacial SA Unit, 6053 Women's and Children's Hospital, North Adelaide, Australia; 4 School of Psychology, 1066 the University of Adelaide, Adelaide, Australia; 5 Discipline of Paediatrics, Adelaide Medical School, 1066 the University of Adelaide, Adelaide, Australia

**Keywords:** team care, cleft lip and palate, retrospective study

## Abstract

**Objective:**

To describe the timing of surgeries, characterize the number of surgical and anesthetic events, and describe the total number and timing of cleft-related and ancillary surgical procedures from birth to late adolescence, stratified by cleft subtype and syndrome status.

**Design:**

Retrospective, cross-sectional clinical review of medical and surgical records.

**Setting:**

A tertiary children's hospital with a dedicated Cleft and Craniofacial Surgical Unit in Adelaide, South Australia.

**Participants:**

Children aged 0 to 18 years who underwent surgical intervention for orofacial clefts at the Women's and Children's Hospital between 1985 and 2021.

**Interventions:**

Surgical management of orofacial clefts and ancillary procedures performed under general anesthesia (GA).

**Main Outcome Measure(s):**

Timing and number of cleft-related surgeries; total number of procedures performed under GA; comparisons by syndrome status and cleft subtype.

**Results:**

A total of 746 children were included. The mean age at primary lip repair was 4.8 months, and palate repair was 18.4 months. Alveolar bone grafting was typically performed at a mean age of 129.4 months. Later procedures included lip/nasal revision and orthognathic surgery. The mean number of GA procedures per child was 3.9 (SD ± 3.3), significantly higher in syndromic children (mean 6.5, SD ± 5.3) than nonsyndromic children (mean 3.4, SD ± 2.3). Noncleft-related procedures, such as grommet insertion (45.6%) and dental treatment under GA (28.1%) added to the total surgical burden.

**Conclusions:**

Children with orofacial clefts experience multiple surgical procedures throughout development, especially those with syndromic diagnoses. These findings reinforce the importance of long-term, multidisciplinary planning and provide data to guide cleft care protocols and family support.

## Introduction

Repair of orofacial clefts commonly occurs in stages. Primary repair typically refers to surgical repair of a cleft lip (CL) or cleft palate (CP) or more rarely single-stage CL and palate repair. Most infants complete their primary repair CL at 3 to 6 months of age^1^ and CP at 6 to 12 months of age.^2^ Infants with Pierre Robin Sequence or other syndromic forms of clefts may have their CP repair a little later.^2^ Surgery to improve speech may be required when children present with speech difficulties, often due to a leak of air between the mouth and nose.^2^ Common surgeries to improve speech include Z-plasty (Furlow), palatal revision, fistula repair, pharyngoplasty/pharyngeal flap, sphincter (orticochea) pharyngoplasty, posterior pharyngeal wall implant, or implant and palatal lengthening using buccal flaps may also be used in selected cases to address speech concerns.^3^ Alveolar bone grafting involves placement of autologous bone into the alveolar cleft to stabilize the dental arch and support eruption of adjacent teeth. It is usually performed when approximately half to two-thirds of root development of the adjacent teeth has occurred, most commonly between 9 and 12 years of age.^4^ Surgeries, including lip/nose revisions and orthognathic surgeries, may be considered when the individual would like to improve the cosmesis of the primary cleft repair or correct any growth constriction. With an array of surgical procedures potentially required, the inpatient burden of care for individuals can be significant, mostly occurring during formative childhood and adolescent years.^5^

A multidisciplinary team approach, including surgeons, cleft nurses, pediatric dentists, orthodontists, speech and language therapists, and more can be involved in the care of individuals with orofacial clefts. Multiple preoperative, operative, and postoperative outpatient appointments and inpatient admissions are an expected part of the orofacial cleft journey. This can be burdensome for the individual as well as the family unit and healthcare system.^5^ The majority of individuals with CL, with or without CP (CL ± P) occur in isolation without cognitive or other craniofacial condition or syndromes.^6^ However, approximately 30% of individuals have other healthcare needs with medical comorbidities or cleft-adjacent surgical requirements. An example of such surgeries would be grommet insertion for otitis media with effusion, a collection of nonpurulent fluid in the middle ear space which is a notable complication of children with CP, frequently requiring surgical intervention.^7^

Although previous studies have documented individual surgical procedures or the timing of selected cleft repairs,^2,4^ few have comprehensively described the full longitudinal surgical trajectory experienced by individuals with orofacial clefts, particularly across all cleft subtypes, from birth through adolescence.^8^ The cumulative number of surgeries, types of interventions, and associated anesthetic events has not been well characterized in a single study. Furthermore, the impact of patient-specific factors (eg, cleft type, comorbidities) on the surgical burden has not been clearly delineated in the literature. A better understanding of these patterns would enable cleft teams and families to anticipate treatment needs, facilitate care planning, and inform health service resource allocation. This retrospective, cross-sectional clinical review aimed to (1) describe the full range of surgical sequences and timings used to repair orofacial clefts; (2) identify patient and clinical factors associated with the number of surgeries and anesthetic events; and (3) describe the total number and timing of cleft-related and ancillary surgical procedures from birth to late adolescence, stratified by cleft subtype and presence of a syndrome.

## Materials and Methods

### Study Design and Sample

Data were collected from a single tertiary children's hospital in Adelaide, South Australia after ethics approval for the clinical audit was obtained through the Women's and Children's Health Network Human Research Ethics Committee (1215A/06/2024). A combination of historical orofacial cleft database, appointment books, and surgical records was used to identify as many individuals as possible that had been seen at the Cleft and Craniofacial South Australia (CCSA) Unit (formerly known as the Australian Craniofacial Unit) at the Women's and Children's Hospital from 1985 to 2021. A review of medical records, including medical and surgical history noted in the individual's public hospital file, was conducted and completed by April 2024.

### Data Collection

Demographic information, including year of birth, sex, Indigenous status, and involvement with Department of Child Protection, was collected. Cleft-related data, included diagnosis of cleft, associated syndrome (there are 500 recognized monogenic or chromosomal syndromes associated with orofacial clefts, were categorized as syndromic; individuals who were nonsyndromic may have had other recorded medical comorbidities, but not a diagnosed syndrome),^9^ age at cleft surgery for primary and secondary repairs in addition to any other related cleft surgeries and total number of general anesthesia (GA) procedures to date (including noncleft-related surgeries) performed at the Women's and Children's Hospital.

### Statistical Analysis

Data were entered into Microsoft Excel and analyzed using IBM SPSS Statistics (version 29). Descriptive statistics were used to summarize demographic information, cleft type, and the number and timing of surgical procedures. Continuous variables, such as age at surgery and total number of general anesthetic events, were assessed for normality and described using means and standard deviations. Categorical variables, such as cleft subtype and presence of syndromes, were reported using frequencies and percentages. Comparative analyses between children with and without syndromic diagnoses were conducted using independent samples *t* tests for continuous variables with normal distribution and χ^2^ tests for categorical variables.

## Results

### Description of Sample

There were 746 children identified as having an orofacial cleft seen within the CCSA unit between 1985 and 2021. Demographic information, including sex, Indigenous status, and care under child protection unit, are described in Table 1. The types of cleft diagnoses are described in Table 2. Diagnostic subtype data were available for 670 children, whereas the overall cohort included 746 children.

**Table 1.** Demographic Information Including Sex, Indigenous Status, and Care Under the Child Protection Unit of the Children With an Orofacial Cleft, Either Syndromic or Nonsyndromic.

	Total (N = 746)		No Syndrome (N = 623)		Syndrome (N = 123)	
Category	N.	%	N.	%	N.	%
Sex						
Male	396	53.2	339	54.4	57	46.7
Female	349	46.8	284	45.6	65	53.3
Indigenous status						
Yes	56	7.8	46	7.7	10	8.2
No	630	88.0	523	88.1	107	87.7
Unknown	30	4.2	25	4.2	5	4.1
Child protection						
Not in child protection	631	89.5	522	89.4	109	90.1
In child protection	33	4.7	25	4.3	8	6.6
Unknown	41	5.8	37	6.3	4	3.3

**Table 2.** Orofacial Cleft Diagnoses Where Diagnostic Subtype Information Was Available for 670 Children, Either Syndromic (115 Children With Subtype Data Available) or Nonsyndromic.

Diagnosis	Total		No Syndrome		Syndrome	
	N.	%	N	%	N	%
CL	89	13.3	87	15.7	2	1.7
CL incomplete	33	4.9	32	5.8	1	0.9
CL bilateral	15	2.2	14	2.5	1	0.9
CL midline	1	0.1	0	-	1	0.9
Microform lip	8	1.2	8	1.4	0	-
CL + alveolus	11	1.6	10	1.8	1	0.9
CL + nose	12	1.8	10	1.8	2	1.7
CL + P (unilateral LHS)	22	3.3	22	4	0	0.9
CL + P (unilateral RHS)	23	3.4	22	4	1	0.9
CL + P (unilateral NOS)	58	8.6	54	9.7	4	3.4
CL + P (bilateral)	39	5.8	33	5.9	6	5.2
CL + P + Nose	15	2.2	15	2.7	0	-
CP	233	34.7	161	29	72	62.1
Cleft soft palate	29	4.3	23	4.1	6	5.2
Cleft uvula	2	0.3	2	0.4	0	-
Submucous	56	8.3	42	7.6	14	12.1
Bifid uvula	3	0.4	2	0.4	1	0.9
Facial cleft (Tessier)	21	3.1	18	3.2	3	2.6
**Total**	**670**		**555**		**115**	

Abbreviations: CL, cleft lip; CL + P, cleft lip and palate; CP, cleft palate.

### Syndromes Associated with Cleft

From all included children, 434 (64%) had some medical diagnosis in addition to having an orofacial cleft. Identified syndromes were presenting for 124 children, and the most common syndromes are described in Table 3. Of these, 115 had diagnostic subtype data available (Table 2), and Table 3 reports the 100 children with syndromes represented more than once in the cohort.

**Table 3.** Syndromes Identified in the Current Cohort of Children With an Orofacial Cleft Where n > 1 (Total 100 Children).

Syndrome	N	%
Pierre Robin	53	7.7
DiGeorge_Velocardiofacial	10	1.5
Sticklers	8	1.2
Goldenhar	5	0.7
Treacher Collins	4	0.6
Kabuki	4	0.6
Hemifacial microsomia	4	0.6
CHARGE	4	0.6
Smith Lemli Opitz	3	0.4
Vander der Woude	3	0.4
Trisomy 21	2	0.3
**Total**	**100**	

### Timing of Cleft-Related Surgery

**Figure 1.**
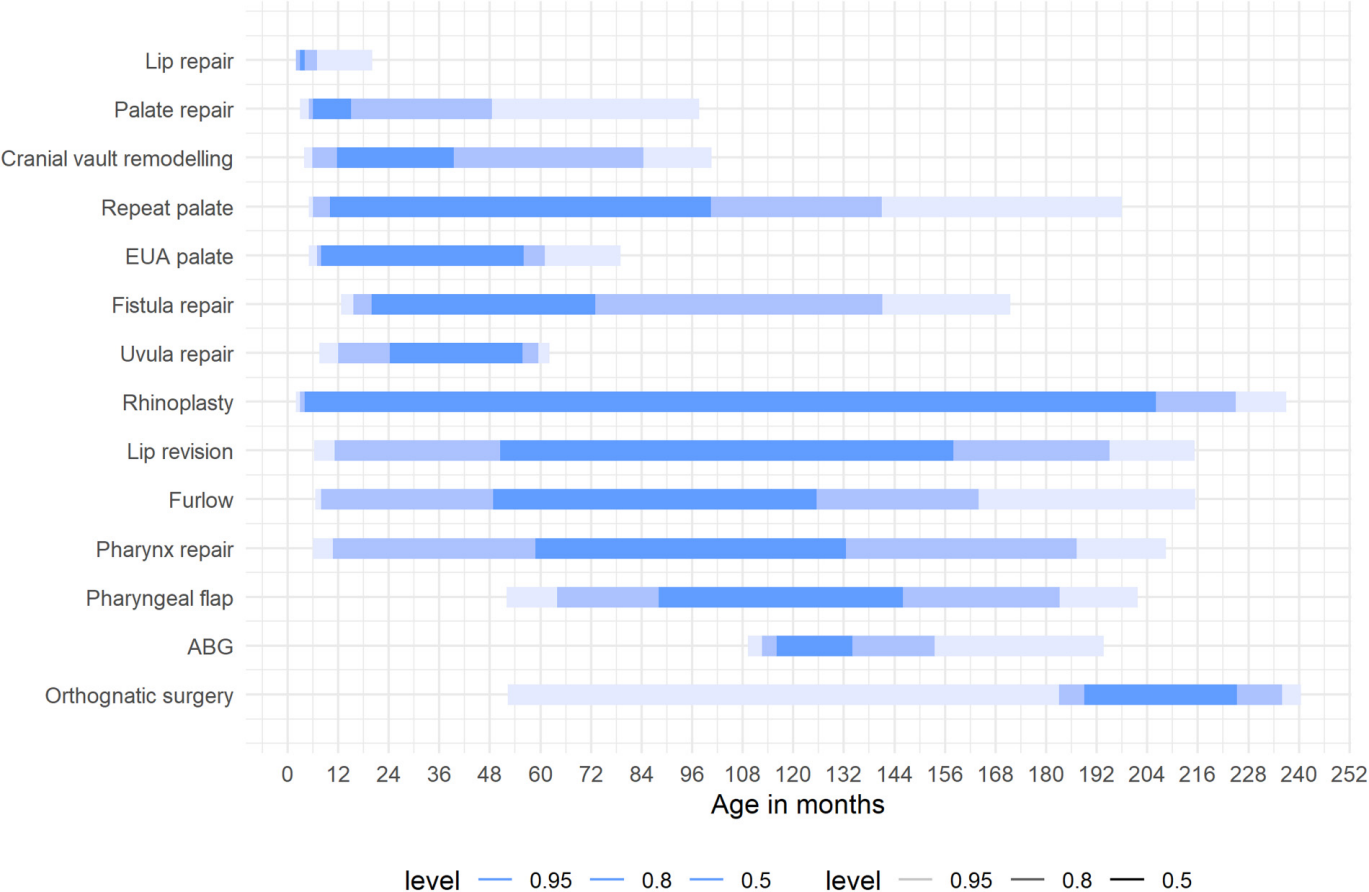
Bar Graph Illustrating the Mean Age (in Months) at which Various Cleft-Related Surgeries were Performed for each Surgery Type. Error Bars Represent Standard Deviations, Indicating Variability in Timing Across the Cohort.

The timing of cleft-related surgical interventions varied according to the procedure type (Figure 1). Primary lip repairs (n = 285) were typically performed at a mean age of 4.8 months (SD ± 7.1). Palate repairs (n = 418) occurred at a mean age of 18.4 months (SD ± 27.0), with procedures such as repeat palatal repairs (n = 76) and palatal fistula repairs (n = 39) performed later, at mean ages of 61.4 months (SD ± 59.7) and 59.6 months (SD ± 49.8), respectively. Surgeries aimed at improving speech, including Furlow palatoplasty (n = 66), pharyngeal flap (n = 5), and general pharynx repairs (n = 30), were performed at mean ages of 86.3 months (SD ± 59.6), 117.2 months (SD ± 61.6), and 96.5 months (SD ± 62.4), respectively. Alveolar bone grafting (n = 98) was most commonly undertaken at a mean age of 129.4 months (SD ± 21.2). Later surgical procedures, including lip revisions (n = 47), rhinoplasties (n = 41), and orthognathic surgery (n = 21), were typically carried out in later childhood or adolescence, with mean ages of 7 years and 10 months (SD ± 5 years and 11 months), 8 years and 2 months (SD ± 7 years and 11 months), and 16 years and 4 months (SD ± 4 years and 2 months), respectively.

### Total Number of Surgeries

**Figure 2.**
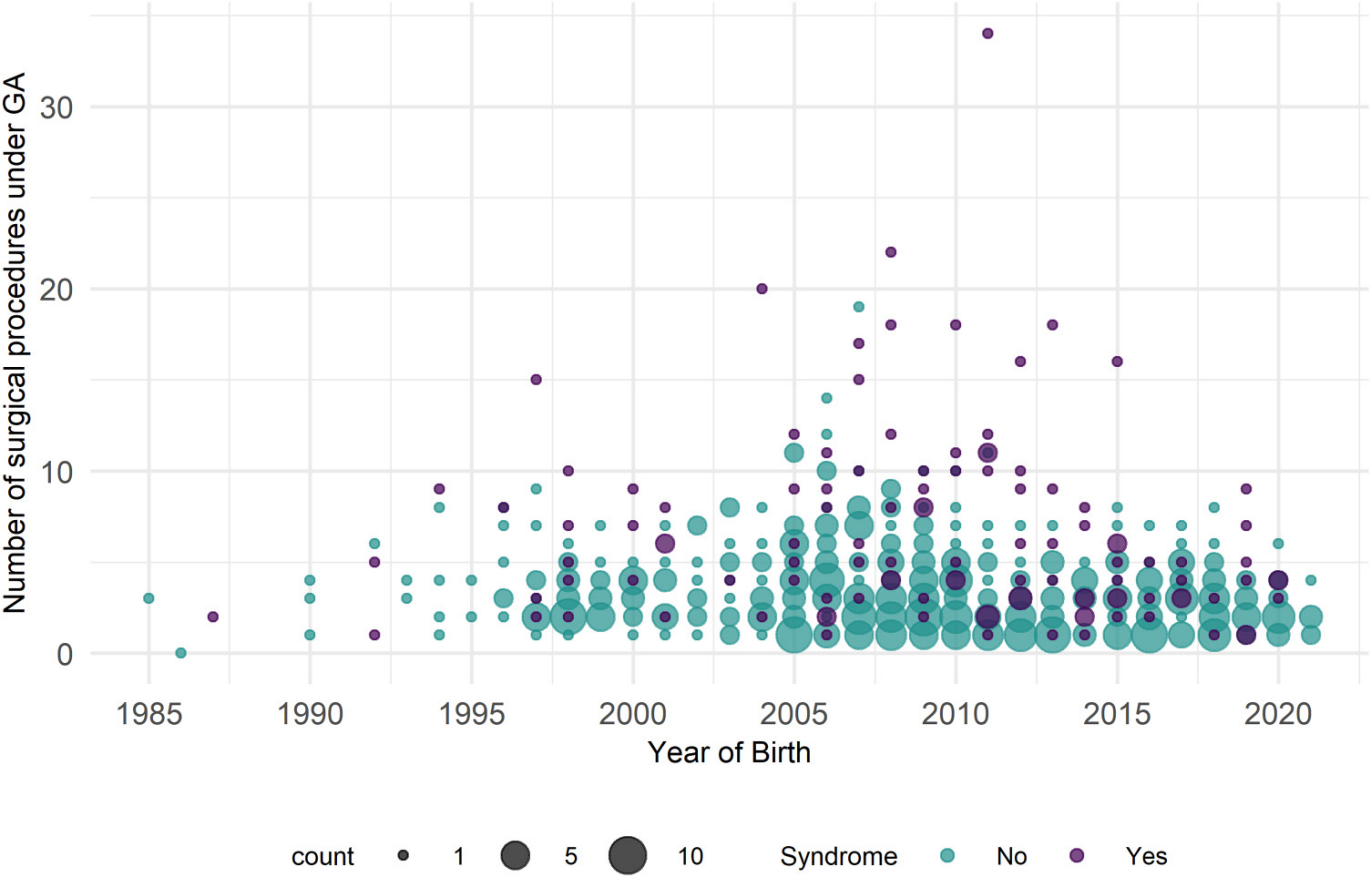
The Total Number of General Anesthesia (GA) Procedures Undergone by Children with syndromic and Nonsyndromic Orofacial Clefts, Plotted Against Age in Months. Data are Stratified by Syndrome Status to Illustrate the Distribution of Anesthetic Exposure Across Year of Birth.

Children with orofacial clefts underwent a wide range of procedures requiring GA (Figure 2). The overall mean number of general anesthetic events per child was 3.9 (SD ± 3.3). This mean reflects the entire cohort, including children with isolated CL or CP who generally undergo fewer procedures compared to those with CL and CP, who often require multiple staged surgeries. Children with a diagnosed syndrome had a significantly higher mean number of GA events (mean 6.5, SD ± 5.3) compared to those without a syndrome (mean 3.4, SD ± 2.3). While 17.8% of children underwent only one procedure under GA, the remainder experienced multiple exposures, with 20.9% having 2, 16.8% 3, and 14.5% 4 GA events. A smaller proportion (4.1%) underwent more than 11 procedures under GA. In addition to cleft-related surgeries, many children had other procedures under anesthesia, including dental treatments (28.1%), grommet insertions (45.6%), and interventions for otitis media (46.3%).

## Discussion

The surgical trajectory observed in this cohort is consistent with international standards recommending primary CL repair within the first 3 to 6 months of life and CP repair by 12 to 18 months of age.^10,11^ While the international standard for palate repair is generally between 9 and 12 months, our cohort had a mean repair age of 18.4 months. This may be influenced by syndromic diagnoses, airway concerns, comorbidities, or health service delays associated with a public hospital setting. However, our data reveal that a substantial proportion of children continue to require cleft-related interventions throughout later childhood and adolescence, including speech surgeries, alveolar bone grafting, lip and nasal revisions, and orthognathic procedures. These extended care needs are well recognized in cleft care pathways globally,^12,13^ but few studies provide such detailed longitudinal data on actual delivery within a public health setting. The relatively low number of bone grafts may reflect that some children had not yet reached the appropriate dental age at the time of data collection. Others may have received the procedure in private settings, particularly if undergoing private orthodontic care. Delays within the public system, including difficulty retaining public orthodontists and long waiting lists, may also have contributed to later-than-expected timing. The relatively low number of rhinoplasties and the younger mean age may be explained by patients seeking definitive nasal procedures in private settings after adolescence, which were not captured in this dataset. The present findings reinforce the importance of long-term multidisciplinary follow-up and sustained service planning extending beyond the early years.^14^

Children with syndromic clefts demonstrated a significantly higher number of GA procedures and surgical events, reflecting the added clinical complexity associated with coexisting anomalies and comorbidities.^15^ This aligns with previous literature reporting increased health service utilization among children with concurrent syndromes.^16^ The average number of procedures reported must be interpreted cautiously, as it does not fully reflect the surgical burden experienced by patients with more complex cleft phenotypes, such as CL and CP, who often require multiple staged interventions, including revisions or orthognathic procedures. While GA is considered safe in pediatric populations, the frequency of repeated exposures, particularly during early neurodevelopmental periods, has prompted ongoing investigation into potential long-term cognitive or behavioral effects.^17^ Although causality remains unproven, these concerns highlight the need for judicious surgical planning, consolidation of procedures where feasible, and informed counseling of families regarding risks and benefits.

In addition to cleft-specific surgeries, the prevalence of noncleft procedures such as grommet insertion, otitis media management, and dental treatment under GA further underscores the systemic nature of care required. These findings support existing evidence that children with CP, in particular, are more susceptible to middle ear pathology, dental caries, and other developmental issues necessitating medical or surgical intervention.^7,18,19^ As per the current protocol through the CCSA, grommet insertion is performed at the time of CP repair wherever possible, ensuring 2 procedures are addressed under a single general anesthetic.^20^ Coordination between surgical teams, audiology, pediatric dentistry, speech therapy, and allied health professionals remains essential for integrated and timely cleft unit care.

The psychosocial impact of the surgical burden should not be underestimated. Recurrent hospitalizations, long recovery periods, and visible differences can affect quality of life, social participation, and educational engagement.^21^ Parents often face logistical, emotional, and financial stress in navigating these ongoing health demands.^22^ Understanding the timing and volume of surgical care can assist teams in setting realistic expectations with families, fostering resilience, and identifying key timepoints where additional psychosocial support may be warranted.^23,24^ This is particularly relevant in adolescence, when appearance-related concerns and social pressures may influence perceptions of treatment outcomes and satisfaction.^25^ As children mature, it is also important to actively involve them in treatment planning and decision-making. Shared decision-making not only supports autonomy but may also improve adherence, psychological well-being, and long-term satisfaction with care.

From a health systems perspective, the findings of this study highlight the resource-intensive nature of comprehensive cleft care, with implications for workforce planning, operating theater availability, and funding allocation. This is consistent with what is known about the overall cost of orofacial cleft care from birth to maturity.^26^ Publicly funded tertiary services, such as the CCSA unit, must be adequately resourced to support the long-term and often complex needs of this patient group.^27^ Furthermore, consistency in care delivery, particularly regarding timing of key interventions, is important for equity across geographical and socioeconomic groups.^28^ These results may inform service evaluation and benchmarking efforts within Australia and internationally. The distribution of cleft subtypes in our cohort differed from many international reports, with relatively higher proportions of CP and lower proportions of CL and CP. This may reflect local population demographics, referral patterns, or variation in case ascertainment over time. These differences should be considered when comparing results across centers.

This study provides empirical support for structured cleft protocols that reflect the extended nature of cleft care. The Cleft Lip and Palate Protocol developed by the CCSA unit provides information for the multidisciplinary service regarding postoperative care following cleft-related surgeries, the pathway for new cleft referrals, information regarding feeding and speech, hearing and dental care, as well as anticipated cleft-surgery timings.^20^ By quantifying the surgical and anesthetic burden across developmental stages, the findings of this study offer important insights for cleft teams aiming to deliver high-quality, coordinated, and patient-centered care. In addition to medical and surgical planning, embedding psychosocial support into cleft care pathways is critical. Families and patients may benefit from integrated access to social work, psychology, and peer support services to manage the emotional, social, and practical challenges associated with long-term treatment. Future research could explore patient-reported outcomes alongside surgical timelines to better understand the lived experience and long-term functional outcomes associated with cleft interventions.

### Strengths and Limitations

While this study draws from the largest public cleft surgical unit in South Australia, not all children born with orofacial clefts in the state may have received their entire treatment at the WCH. However, the WCH houses the only dedicated craniofacial unit in the state, established in 1975 as the Australian Craniofacial Unit, and served as the primary referral center for South Australia and the Northern Territory. A small number of patients may have traveled from overseas for treatment; as this information was not consistently recorded, all individuals were included in the analysis. The findings are most generalizable to the South Australian population but are likely applicable to other Australian states and territories with similar service structures. It should also be noted that surgical protocols and techniques evolved between the 1980s and 2020s, which may contribute to variation in timing and types of procedures observed across the study period.

Some individuals may have sought cleft-related care through private providers; however, the availability of publicly funded, comprehensive multidisciplinary care through the CCSA is likely to have captured most cases. Surgical records used in this study are maintained through multiple sources, including theater logs, preoperative nursing documentation, and procedural notes, ensuring high reliability of surgical data. Surgical timing was calculated from the date of birth rather than corrected gestational age. While this may introduce minor variability in infants born prematurely, the impact on surgical timing classifications is expected to be minimal.

As a retrospective audit, the study is limited to objective clinical documentation. It does not capture the reasons for revision surgeries or patient- and family-driven decisions, such as those influenced by aesthetic concerns or functional preferences. Additionally, the study focuses solely on inpatient surgical care. Outpatient appointments and nonsurgical components of cleft management, such as speech therapy, dental and orthodontic care, psychosocial support, and nursing follow-up, were not consistently available across the study period and thus could not be reliably reported. As a result, the full scope of cleft-related healthcare utilization and associated financial, emotional, and time-related costs is likely underestimated. Although cleft subtypes were recorded, the number of patients in each subgroup was insufficient to permit statistically meaningful analyses of surgical burden by cleft type. For this reason, we reported aggregated outcomes across the cohort. This approach provides a robust overview of the surgical burden, but we recognize that it limits direct comparison with centers reporting larger, subtype-specific cohorts. Furthermore, the dataset does not capture individuals who transition to adult services after the age of 18. Surgeries such as orthognathic procedures, secondary lip and nasal revisions, often performed at the Royal Adelaide Hospital or in private practice, may not be reflected in this study. Some patients had not reached skeletal maturity at the time of data collection, others may have undergone orthognathic surgery in private practice not captured in our dataset, and some may have chosen not to proceed. While it is possible that effective primary management reduced the proportion requiring orthognathic intervention, this cannot be confirmed from our data. This may result in a slight underestimation of the total surgical burden for some individuals.

## Conclusion

This 36-year retrospective review of cleft surgical care in South Australia provides one of the most comprehensive longitudinal accounts of surgical timing, frequency, and burden among children with orofacial clefts within a public health system. The findings highlight the prolonged and multifaceted nature of cleft treatment, particularly among syndromic cases, with significant implications for families and healthcare services. By documenting the timing and frequency of cleft-related and ancillary surgical interventions, this study reinforces the need for coordinated, multidisciplinary, and long-term cleft care planning. These data, drawn from 36 years of service provision in a single public cleft unit, provide benchmarks for clinicians, policymakers, and service planners aiming to improve cleft protocols, ensure equitable access to care, and support families through what is often a complex and extended healthcare journey. Future research should incorporate patient-reported outcomes and explore the broader psychosocial and economic impacts of cleft treatment across the lifespan.
